# A review of the continuous professional development system for pharmacists

**DOI:** 10.1186/s12960-021-00700-1

**Published:** 2022-01-06

**Authors:** Jorge P. B. Batista, Carla Torre, José Manuel Sousa Lobo, Bruno Sepodes

**Affiliations:** 1 Ordem dos Farmacêuticos (Portuguese Pharmaceutical Society), Lisboa, Portugal; 2 Unidade de Saúde Pública Internacional e Bioestatística, Instituto de Higiene e Medicina Tropical, Universidade NOVA de Lisboa, Lisbon, Portugal; 3 Faculdade de Farmácia da Universidade de Lisboa, Department of Pharmacy, Pharmacology and Health Technologies, Lisbon, Portugal; 4 Laboratory of Systems Integration Pharmacology, Clinical and Regulatory Science - Research Institute for Medicines of the University of Lisbon (iMED.ULisboa), Lisbon, Portugal; 5 UCIBIO-Applied Molecular Biosciences Unit, MedTech, Laboratory of Pharmaceutical Technology, Department of Drug Sciences, Faculty of Pharmacy, University of Porto, Porto, Portugal

**Keywords:** Pharmacists, Pharmacy, Continuous Professional Development, Accreditation, Pharmacy education

## Abstract

**Background:**

The Portuguese Pharmaceutical Society (PPS) implemented a system of Continuous Professional Development (CPD) for pharmacists in 2004. This system has evolved throughout the years, and currently all active pharmacists in Portugal are required to participate in the CPD program. Each CPD cycle takes 5 years. In each cycle, pharmacists must collect 15 CPD points, through participation in educational activities. The PPS accreditation process is managed via an online platform, where education/training providers, as well as pharmacists themselves, can submit educational activities for accreditation. Pharmacists may access their CPD status and assess their development at any point. The objective of this study was to analyze and review the educational activities submitted by providers over a 11-year period (2009–2019).

**Methods:**

Data from activities were retrieved from the PPS CPD online platform. All educational activities were labeled according to the area of pharmaceutical professional focus, type of promoter, and activity type.

**Results:**

During the study 3685 activities were analyzed. Over the last decade, submitted activities for accreditation increased in 52.6%. A significantly high proportion (98.9%) of these activities has been accredited. Promoters of activities were mostly pharmacies sectoral associations (29.6%), consultancy/training companies (19.6%), the PPS (18.5%), pharmaceutical industry (17.7%) and wholesalers’ consortia (9.0%). Academia represented only 2.3% of the total amount of educational activities. The most frequent topics were related to “pharmacology & pharmacotherapy” (9.9%), followed by “counselling” (9.8%) and “management & administration” (7.2%). The most accredited type of activities was face-to-face (68.9%) and e-learning trainings (13.1%).

**Conclusions:**

This study shows increasing interest in submitting CPD activities for accreditation between 2009 and 2019, but it also demonstrates that Academia could play a more interventive role in the lifelong learning education of Portuguese pharmacists.

**Supplementary Information:**

The online version contains supplementary material available at 10.1186/s12960-021-00700-1.

## Background

Pharmacists are healthcare professionals whose professional responsibilities include, among others, ensuring that people derive maximum benefit from their pharmacological and non-pharmacological therapeutic interventions. This requires pharmacists to keep up to date with the latest developments in pharmacy practice, pharmaceutical sciences, professional standards requirements, laws, and regulations governing pharmacy and medicines, and the advances in scientific knowledge and technology relating to the use of medicines. It is widely accepted that this can only be achieved by an individual’s personal commitment to Continuing Professional Development [1].

The rapid development of science and patient safety issues require pharmacists to maintain their competencies updated to the existing knowledge and evidence. In this regard, implementing Continuing Professional Development (CPD) programs is considered to be of the utmost importance [2]. The International Pharmaceutical Federation (FIP) has adopted the concept of CPD in 2002 and defined CPD as *“the responsibility of individual pharmacists for systematic maintenance, development and broadening of knowledge, skills and attitudes, to ensure continuing competence as a professional, throughout their careers”* [1].

This is intended to be an on-going cyclical process built over different fundamental pillars: self-appraisal, development of a personal learning plan, taking action or implementing the learning plan, and evaluation [3].

With the rapid progress observed in the profession of pharmacist—namely due to the discovery of new medicinal products, advancements in clinical practice, and the ever-expanding role of pharmacists’ intervention in the healthcare system—the relevance and importance of post-graduate education plays an important and more definitive role. CPD is responsible for knowledge increase, changes of practice and boosts professional development [4].

A report funded by the European Union (EU), with the aim of reviewing and mapping the different CPD and lifelong learning programs for healthcare professional in the EU and European Free Trade Association (EFTA) countries, showed that a mandatory CPD system for Pharmacists was found to be in place in 20 out of 31 countries, whereas voluntary CPD or no CPD system at all was still found in the rest of the countries. Most systems are based on a number of credits, hours, activities, or learning outcomes with respect to skills, knowledge and competencies required [5].

A report published by FIP, regarding different CPD systems in the world, revealed that mandatory CPD among pharmacists has been led by some countries, in which the pharmacy practice is also most developed—Portugal included. Other flagship CPD systems of reference have been found all over the world, namely in Australia, Canada, China, Ireland, Japan, Malaysia, New Zealand, Singapore, Spain, United Kingdom and the United States of America [6].

The regulation of pharmacist’s profession includes registration in a professional body, subject to internal regulations, and the fulfillment of several orienting principles and professional and ethical guidelines. The Portuguese Pharmaceutical Society (PPS) regulates the pharmacist’s profession in Portugal. After registration in the PPS (subject to recognition of professional qualification), pharmacists are automatically enrolled in a Continuous Professional Development program (CPDp), managed by the PPS and enacted in the Statutes (bylaws) of the Society [7].

The model for admission and qualification of Portuguese pharmacists was launched in Portugal in 2004, foreseeing a mandatory renewal of the professional license linked with the collection of 15 CPD points, which were granted on a basis of 0.1 CPD points per hour of training/learning [8].

In 2009, following an analysis of the system in place, a thorough change was implemented by the National Board of the PPS, which enforced the new CPDp in a format that was known until today. This period marked a consolidation of the CPD system as designed for pharmacists in Portugal [9].

Each CPD cycle lasts for 5 years. During these 5 years, pharmacists must collect at least 15 CPD points, and these points can be collected up to 2 CPD points per year of practice (1 CPD point per semester), amounting to a maximum of 10 collectable CPD points per professional practice activity. The remaining 5 CPD points must be collected through the active participation in educational activities, such as congresses, conferences, training activities, among other, subject to specific regulation [9, 10]. These activities must be previously accredited by the PPS, through an accreditation process that is managed via an online platform (CPDp e-platform). The providers of educational activities must submit a proposal in the e-platform, which is then evaluated by the PPS staff regarding the program duration, speakers’ details, scope, area of practice, among other criteria. Similar systems have been found in other countries, such as Germany, Australia, USA, United Kingdom, Ireland, among others [11, 12].

Pharmacists can also submit their own individual activities for evaluation and accreditation, as long as these have not been yet accredited by the PPS (e.g., internal company training, mentoring of students’ internship, presentation of a research poster/research paper, among other activities). Both educational activities submitted by stakeholders and CPD providers, and pharmacists’ individual activities, are analyzed by the PPS staff (comprised of administrative assistants and dedicated pharmacists), and by the Qualification and Admission Council. This Council is an independent expert panel composed of professionals from different pharmacy specialization areas and academics, who are responsible for the evolution and development of specializations, competency development and CPD system.

At any moment in time, pharmacists may access their CPD status and assess their professional development. After 17 years of experience of a national Continuous Professional Development program for pharmacists, with over 10 years of consolidated experience, there is still a lack of data to promote an evaluation of the program and its continuous development. Reviewing the CPD program is important to promote the evolution of this tool, to foster stakeholder involvement and to have a quality control and evaluation process, aiming at reinforcing its actions and sharing best practices.

Against this background, a study was conducted to perform a review of the educational activities submitted by education and training providers and accredited over a 11-year period (2009–2019) in the Continuous Professional Development program of the Portuguese Pharmaceutical Society.

The study aimed at reporting the trends in training during this period, evaluating the main areas of interest in CPD for pharmacists and its characteristics, assessing principal stakeholders involved, and evaluating the implementation of the system over a 11-year period, thus exploring drivers, strengths, and bottlenecks of a mandatory system for all practicing pharmacists in Portugal.

## Methods

### Data collection and variables

Data from 3800 educational activities were retrieved from the Portuguese Pharmaceutical Society CPD online platform using Microsoft Excel 2019, covering the period 01/01/2009–31/12/2019. This database contains all the educational activities sponsored by stakeholders. This study comprised only educational activities delivered by stakeholders and did not include individual submissions from pharmacists.

Data extracted consisted of: (i) the year in which the educational activity was evaluated; (ii) the name of the activity’s promoter; (iii) the name of the activity submitted to accreditation; (iv) the outcome of PPS evaluation (non-accredited/accredited); (v) the presence or absence of evaluation within the activity (with evaluation/without evaluation); (vi) the number of CPD points attributed; (vii) the classification regarding the type of activity (face-to-face training, e-learning/b-learning training, conference, congress, post-graduate courses, master’s and PhD degrees, research study, position held in professional organizations and Specialist title); (viii) the activity’s duration (from 1 hour (h) to 5 years); (ix) the total number of participants registered, and; (x) the total number of times that each activity took place in each year (defined as “actions”).

The authors considered a “conference” as an educational event of up to 8 h (1 day) and a “congress” as an educational event with a length over 8 h (more than 1 day). All activities which included a non-face-to-face interaction were considered distance learning/e-learning activities. “Post-graduations courses” were admitted as 1-year length courses, “master’s degrees” as 2-year academic programs, and “PhD” and “Specialist titles” were ranked as 5-year-long programs. Regarding the participation in “research studies” as educational activities (e.g., recruiting patients to pharmacoepidemiology studies in community pharmacy context), a mean-value of its total duration was fixed, since in each study pharmacists could have been involved in different extents and roles.

An activity was considered as “accredited” when CPD points were attributed. Activities were not accredited if they did not comply with the professional scope, were not relevant to professional development or were purely based on commercial information.

Activities could be granted a minimum of 0.075 CPD points (which corresponded to 1 h of training/learning) to a maximum of 15 CPD points (which corresponded to a Specialist title or PhD).

### Data quality and validation

Each education/training activity was screened individually by 2 independent pharmacists, and data were analyzed separately. If information was missing from the database, a search through paper registries was conducted to complete as much as possible all information, and subsequently double-checked for accuracy by an external reviewer (pharmacist).

After this step, all educational activities with one or more fields of missing/invalid information, or duplicate entries were excluded.

### Data management and analysis

All educational activities were classified regarding the type of promoter and the geographic scope (Additional file 1: Annex S1). All activities were also labeled by area of professional focus, up to 3 labels per activity, within a range of areas (Additional file 1: Annex S1). As this process has been automated in the e-Platform since 2017, all the activities until that year were manually labeled and reviewed. Each activity was reviewed individually by 2 pharmacists and labeled by each pharmacist independently. Any disagreements to the analysis were resolved by consensus. Whenever consensus was not possible, an external pharmacist acted as judge and screened all selected activities to confirm their inclusion.

Information retrieved was anonymized to comply with data protection regulation.

Descriptive analysis was conducted for all study variables. Discrete variables were summarized by absolute and relative counts. Continuous variables were summarized using central tendency measures and dispersion, i.e., mean, and standard deviation (SD), median and inter-quartile range (IQR). A linear regression model was set up to investigate the trends in analyzed activities over the study period. Statistical significance was set at *p* < 0.05. Data were analyzed using a statistical analysis software (IBM SPSS Statistics version 27.0.1.0).

## Results

**Fig. 1:**
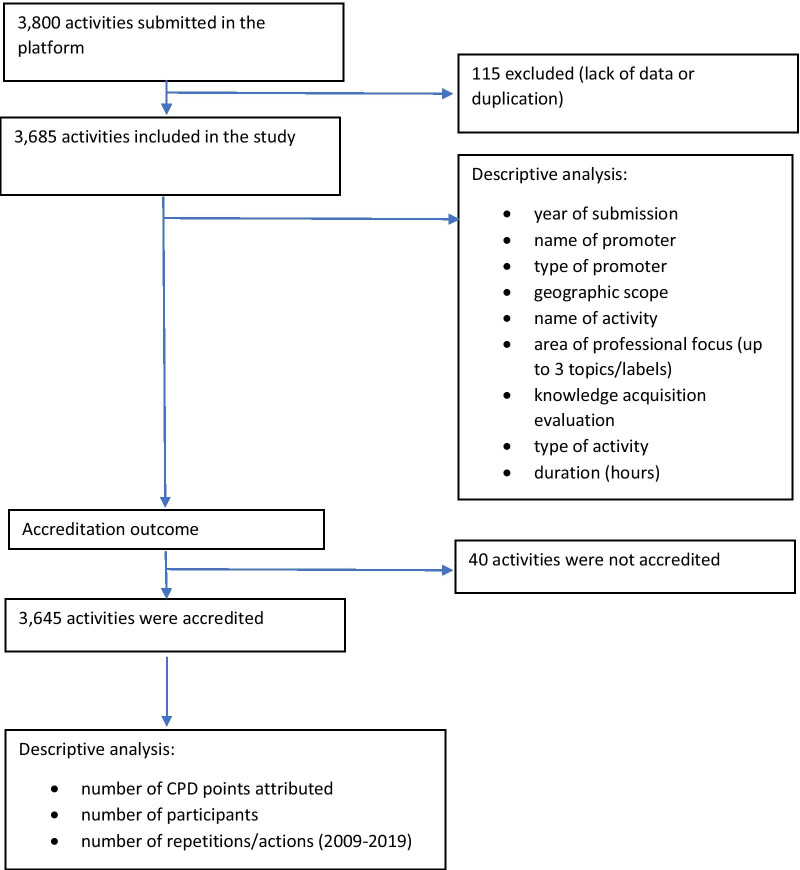
Flowchart of activities analysis

Over the study period, 3800 activities were submitted in the platform by providers, 115 (3.0%) of which were eliminated, due to missing data or duplication of entries. In total, 3685 activities were analyzed (Fig. 1).

Activities were labeled concerning the area of professional focus. Each activity was labeled with a maximum of 3 topics. Of the total of 3685 activities analyzed, 50.9% of activities (*n* = 1875) were labeled with only 1 label, 40.1% (*n* = 1479) with 2 labels and 9.0% (*n* = 331) using 3 labels.

The evolution of activities, participants, actions, and promoters in the period 2009–2019 is summarized in Table 1.

**Table 1:** Activities, participants, actions, and promoters’ evolution in the period 2009–2019

Year	Analyzed activities *n*	Accredited activities *n* (%)	Individual promoters	Number of participants	Number of educational actions^a^	Number of educational actions per 1000 pharmacists registered in the PPS^b^
2009	308	286 (92.9%)	54	20,072	982	85.1
2010	320	320 (100%)	64	29,838	1061	88.7
2011	285	282 (99.0%)	66	27,546	1329	108.3
2012	271	271 (100%)	59	23,948	1467	114.5
2013	282	282 (100%)	70	34,726	1161	89.0
2014	319	319 (100%)	89	41,829	1761	131.8
2015	405	405 (100%)	98	57,272	2221	162.6
2016	311	311 (100%)	88	45,472	2897	205.8
2017	353	352 (99.7%)	107	34,795	3327	230.7
2018	361	361 (100%)	107	31,110	3211	216.6
2019	470	456 (97.0%)	106	42,742	3273	215.7
Total	3685	3645 (98.9%)	310	389,350	22,690	N.A

^a^Each accredited activity could be repeated more than once

^b^PPS, Portuguese Pharmaceutical Society

### Evolution of the CPD program throughout the decade

**Fig. 2:**
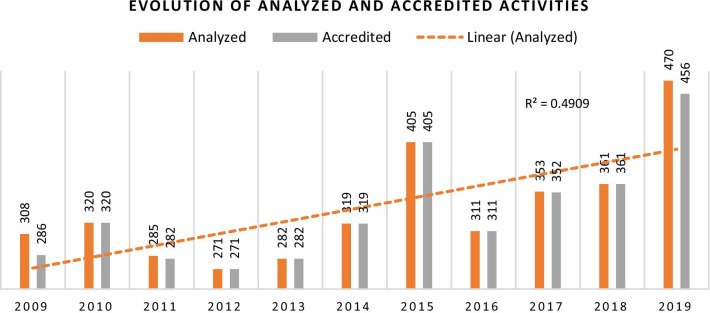
Evolution of analyzed and accredited activities

There was a significant increase (*p* < 0.05) of 52.6% in the number of analyzed activities for accreditation over the last decade (308 in 2009 to 470 in 2019) (Fig. 2). A significantly high proportion (98.9%, *n* = 3645) of the analyzed activities have been accredited, as seen in Table 1.

**Fig. 3:**
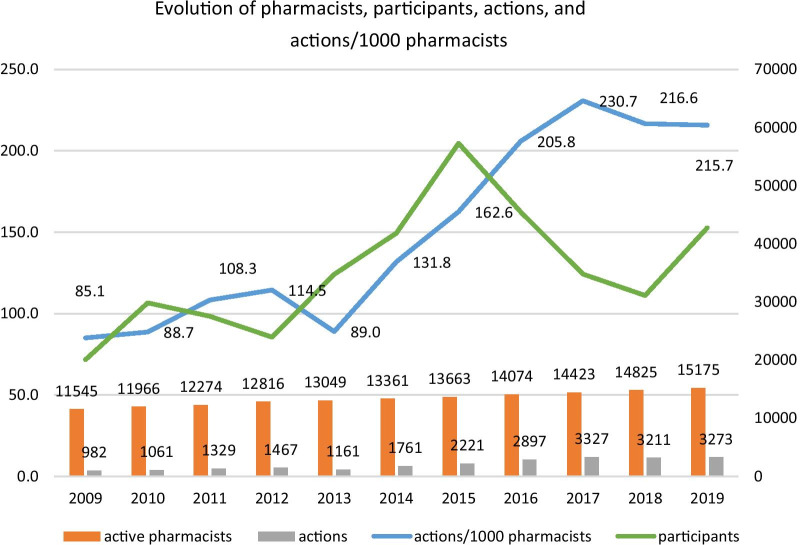
Evolution of pharmacists, participants, actions, and actions per 1000 pharmacists

Throughout this period, as seen in Fig. 3, the number of active pharmacists registered in the PPS increased as well (31.4%, from 11,545 in 2009 to 15,175 in 2019).

We can identify a steady increase in the number of activities that took place each year, in an increase of 233% when comparing the time period (Fig. 3).

During the 11-year period (2009–2019) 389,350 participants took part in the educational activities accredited by the Portuguese Pharmaceutical Society. Although not linear, there is a clear increase (113%) in the number of participants per year. On average, each activity had 106 participants, which represents an average of 35,395 participants per year (Fig. 3).

Data suggest that the number of actions increased throughout the years. Actions are the number of events/repetitions of the accredited activities, i.e., each accredited activity can take place multiple times, each being counted as an action/event. However, even though the overall number of participants increased through time, after 2016 the number of actions per 1000 pharmacists was greater (Fig. 3).

The most common activities were face-to-face trainings (68.9%, *n* = 2538), followed by e-learning training (13.1%, *n* = 585). Conferences (7.9%, *n* = 290) and congresses (5.7%, *n* = 209) ranked 3rd and 4th most common activities.

**Fig. 4:**
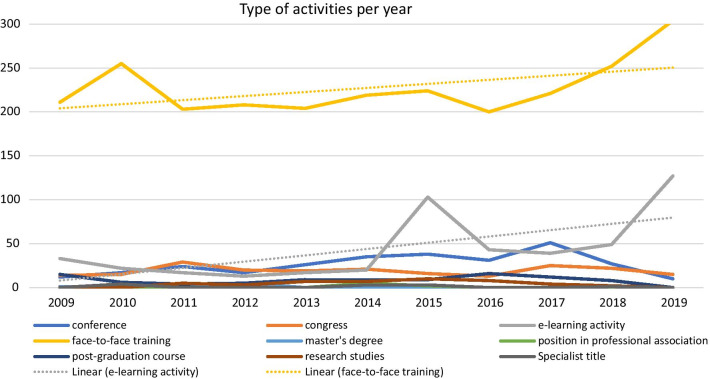
Evolution of type of activities per year

The number of face-to-face trainings, being the most common type of activity, has been stable until 2016. After this point, we can denote a steep increase. With regard to e-learning activities, they have been quite scarce up until 2014. In 2015, there is a peak of e-learning activities, and in the following years we can see a sustained growth, with another peak in 2019. The tendency is for e-learning activities to grow more than face-to-face activities (in what was still a pre-pandemic period). Data suggest that throughout the years “conferences” have risen in numbers, as well as “congresses” (Fig. 4).

### Promoters and stakeholders involved

The promoters of activities analyzed for accreditation were mainly professional and sectoral associations (29.6%, *n* = 1092), consultancy/training companies (19.6%, *n* = 721), the Portuguese Pharmaceutical Society (18.5%, *n* = 681), the pharmaceutical industry (17.7%, *n* = 652), and wholesalers’ consortia (9.0%, *n* = 332). These 5 categories of educational activities’ promoters provide for almost 95% of the total amount of activities analyzed between 2009 and 2019 (94.4%, *n* = 3478). Academia was responsible for a small number of accredited activities (2.3%, *n* = 84).

The database retrieved 310 different promoters, that submitted educational activities throughout the 11-year period (Table 1).

When comparing the number of individual promoters per year, the distribution matches the distribution of activities analyzed for each year. This suggests that the number of promoters who have submitted activities has been increasing throughout the years, and this growth has been concurrently with the number of newly accredited activities (Table 1).

### Activities profile

Most activities accredited were targeted to a national audience (94.2%, *n* = 3471), while a minority (5.8%, *n* = 214) was intended for an international audience. Out of 214 international activities accredited, half (50.9%, *n* = 109) were organized by professional and sectoral associations.

The 10 most common topics (which amount for a total of 56.1% of cases) were “pharmacology & pharmacotherapy” (9.9%, *n* = 577), “counselling” (9.8%, *n* = 572), “management & administration” (7.2%, *n* = 421), “pharmaceutical sciences” (5.1%, *n* = 298), “soft-skills” (4.4%, *n* = 258), “healthcare & wellbeing” (4.2%, *n* = 245), “health education and promotion” (4.2%, *n* = 244), “sales & marketing” (4.1%, *n* = 238), “quality assurance” (3.9%, *n* = 229), and “hospital pharmacy” (3.2%, *n* = 188).

**Fig. 5:**
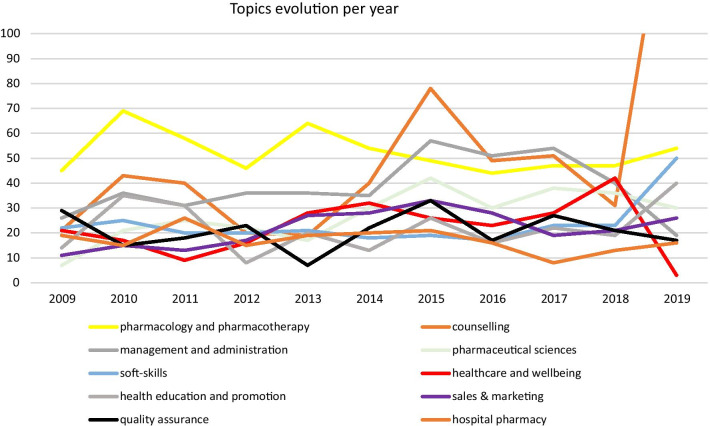
Topics evolution per year

By the analysis of data, we identified that scientific training (labeled as “pharmacology & pharmacotherapy”) has been stable and the most prominent throughout the decade. This has been the most common topic until 2014, with peaks in 2010 and 2013, but with a slight decrease between 2013 and 2016. Furthermore, we can see an increase of training related to “counselling” in community pharmacy, starting in 2013, reaching a peak in 2019. Trainings on the “management & administration” topic increased between 2014 and 2017. Training related to “soft-skills” had a steep increase in 2019, when compared to previous years. Training related to “sales & marketing” has been growing throughout the years, reaching a peak in 2015 (Fig. 5).

Of those activities that were analyzed, more than half (57.5%, *n* = 2119) comprised a knowledge assessment in the program (“evaluation”). This was mostly done through a test/exam taken at the end of the session.

Analyzed activities (*n* = 3685) varied widely in terms of duration. These could range from 1 h (face-to-face or online-training), to 2 years (such as master’s program), or even 5 years (such as PhD or the Specialist titles).

When analyzing activities that took less than a year to complete (*n* = 3662; 99.4%), almost half (48.6%, *n* = 1780) were either 2-h (13.9%, *n* = 512), 3-h (10.0%, *n* = 367), 4-h (9.4%, *n* = 345), 7-h (7.7%, *n* = 282) or 8-h (7.4%, *n* = 274) long.

Activities that can be completed under a day of work (or preferably half a day) are often the most attractive for pharmacists, since they register a high number of participants.

Regarding accreditation with CPD points, the median value was 0.6 and Inter-quartile Range was 0.9. Data suggest that from all accredited activities (*n* = 3645), the great majority (*n* = 3628; 99.5%) were granted between 0.075 and 5 CPD points (Table 2).

**Table 2:** Attribution of CPD points per type of activity

	Conference	Congress	e-learning training	Face-to-face training	Master’s degrees	Post-graduate course	Research studies on pharmacy practice	Specialist titles
Median	0.300	0.850	0.800	0.525	10.000	5.000	0.375	10.000
IQR	0.210	0.600	2.200	0.760	0.000	0.100	0.830	5.000
Mean	0.318	1.033	1.554	0.806	9.286	4.681	1.023	12.272
Std. error	0.012	0.049	0.074	0.019	0.714	0.077	0.210	0.787
Minimum	0.075	0.200	0.075	0.075	5.000	0.500	0.100	10.000
Maximum	2.100	3.800	5.000	5.000	10.000	5.000	5.000	15.000

**Fig. 6:**
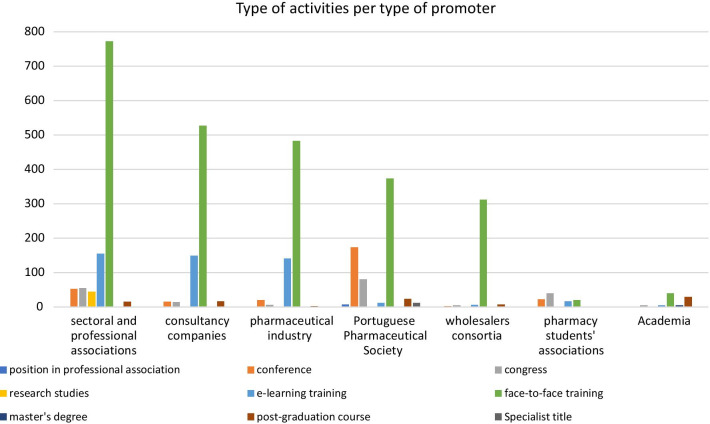
Type of activities per type of promoter

Face-to-face trainings were mostly organized by sectoral and professional associations, consultancy/training companies, the pharmaceutical industry, the Portuguese Pharmaceutical Society, and wholesalers’ consortia. E-learning training was mostly organized by the pharmaceutical industry, consultancy/training companies, and sectoral and professional associations. Most conferences and congresses were organized by the Portuguese Pharmaceutical Society, and some were organized by sectoral and professional associations and students’ associations. Specialist titles and positions held in professional associations were solely awarded by the PPS. Post-graduation courses and master’s degrees were organized mostly by Academia, the former being also organized by sectoral/professional associations, consultancy, and the PPS (Fig. 6).

## Discussion

The study shows a significant increase in the interest of CPD activities submitted for accreditation of the Portuguese Pharmaceutical Society throughout the period 2009–2019 (increase of 52.6%). Apart from the interest shown by promoters in accrediting activities for pharmacists, there is also a noticeable increase in both the number of activities that took place throughout the years and the number of participants, which are consistent with published literature [13]. Interestingly, even though the number of active pharmacists increased in Portugal steadily, the number of actions per 1000 pharmacists increased greater, which represents widely available opportunities for pharmacists’ lifelong learning. CPD activities became widely available for pharmacists, increasing the chances of practice improvement and advancement [14]. Participation in activities of professional development accredited by a Professional Regulator is highly valued by pharmacists, as both quality assurance is warranted, and certification helps to build knowledge and professional confidence [15]. Accreditation of activities guarantees that CPD opportunities meet technical-scientific criteria and provide assurance to the content quality [12].

### How pharmacists adapted to adverse conditions

Most common topics reveal that pharmacists are interested in getting updated with the changes in new and innovative medicinal products and refreshing their knowledge of pharmacotherapy topics, which is consistent with published data [16, 17]. However, we can already see a shift towards activities focusing on patient counselling and management & administration [18]. This is mostly noticeable after 2015. As the majority (60%) of pharmacists work in community pharmacy, their work is often related to administrative and management roles. This quite often comprises financial management of pharmacies and pharmacy services development. Nonetheless, the shift seen towards more involvement in training related to managerial and financial skills could be also a response of adaptation to a sector severely affected by the financial crisis.

Firstly, the financial crisis in Portugal had a severe impact in the healthcare sector, and community pharmacies were no exception [19, 20]. The results of this study, namely the increased interest in training in areas of management and administration, are in line with the need that community pharmacists felt to expand their role as managers of private-owned community pharmacies and to develop professional skills on management and administration. The economic crisis years in Portugal resulted in a subsequent effect of financial crisis. Troika austerity measures that were implemented in the healthcare sector—and especially in pharmaceutical expenditure—led to an augmented interest of pharmacists in capacity building opportunities to increase their knowledge and skills in management and administration [20]. Due to the restrictions imposed by Troika, and the general financial crisis that affected Portugal, pharmacists had to dedicate more of their roles in the management of community pharmacies. For that, it is clear that pharmacists felt the need to develop management skills, which contributes to explaining the increase observed in these types of activities.

Secondly, expanded interest in training related to sales and marketing, as well as soft-skills, can be encompassed by the expanded role pharmacists have been developing, mainly in community pharmacies. In the ever-expanding scope of practice, pharmacists have been focusing more on patient counselling and service provision [21]. Developing an active pharmacy workforce leverages tools to increase productivity, growth and sustainability [22].

Finally, Gregório et al. described the recent interest that community pharmacies have taken upon new services and pharmacists involvement in new pharmacy services [23]. Policarpo et al. have also discussed how pharmacy services are perceived by population, in an ever-increasing demand by the public [18]. The implementation of new pharmacy services, linked with patient counselling, is preceded by an implementation of training and professional development to train pharmacy staff, which is consistent with the study data.

### Digital transformation

Face-to-face trainings were the most common types of CPD activities, especially if they were under one day of duration. This information is consistent with the published literature [15, 17]. However, the increased offer of digital opportunities led for a change in the learning paradigm, with e-learning activities being increasingly more present in the market. These 2 types of formal training continue to be the most relevant and the most chosen by Portuguese pharmacists, according to the data presented in this study.

A protocol signed between the PPS and an e-learning training international institute resulted in a peak in the offer of e-learning activities accredited in 2015 [24, 25]. This was pertinent to increase the offer of training in Portuguese language for pharmacists.

Even though there is growth in both types of learning, data suggest that e-learning opportunities increased more than traditional face-to-face training, and more opportunities are rising in the market. As most Portuguese pharmacists are considered to be young (41% is 35 years or younger, and 72% is 44 years or younger), we expect to see an increase in the number of e-learning activities in the future. Quite often young pharmacists prefer innovative technologies for learning, while more experienced pharmacists enjoy the face-to-face connection and the opportunity to network [13, 26].

### Academia lags behind in CPD performance

The study found that private initiatives (professional and sectoral associations, consultancy/training companies, pharmaceutical industry, wholesalers’ consortia) and the PPS itself contributed the most for organizing CPD activities. Surprisingly, Academia ranked one of the least proactive stakeholders in organizing CPD activities, being at times surpassed even by students’ associations. One must acknowledge, however that in most cases, these student’s associations or junior start-ups are sponsored and enjoy privileged orientation from Academia. These findings were unexpected, as they contrast to what has been described in the literature [13].

Comparing all promoters of CPD activities, data suggest that Academia is somehow lagging behind. Activities such as congresses, short post-graduation courses and conferences could be further delivered by Academia, apart from more master’s degrees and PhD Degrees. Academia plays a role in research and knowledge development, and has sufficient manpower and experts in the topics to organize self-sustainable programs that could impact positively the opportunities for Continuous Professional Development of pharmacists [27]. Published data elsewhere, shows a deeper involvement of Academia in the contribution of Continuous Education and Continuous Professional Development, namely in Canada, Australia, the United Kingdom and other European countries [15, 28]. As independent bodies that promote both education and research, universities are placed in a prestigious position to bridge the gap between Academia and practice environments. This often reflects an unique opportunity to translate knowledge into practice [29]. Pharmacists’ uptake to CPD and involvement as part of lifelong learning should be nurtured and embedded in the pharmacy curriculum, thus emphasizing the importance of it and motivating professionals to include it as part of their role as caretakers and service providers [30].

The authors suggest that there is still much room for improvement on Academia to play a more interventive role in the lifelong learning education of pharmacists, as corroborated elsewhere [27].

### Reflections and future development

The study found no link between CPD and the professional area of practice, which confirmed prior experiential data. After a period of reflection and public consultation with Portuguese pharmacists, a new proposal of the PPS Internal Regulation was approved by the end of 2018, published in January 2019, and put into practice in 2020. The revision encompassed adjustments of the CPD program. The changes mainly cover the difference between “core” and “non-core/satellite” activities which pharmacists are granted credits for (being the former areas directly related to professional practice, and the later areas not directly related to pharmaceutical profession), and an adaptation of the system so it reflects better the continuity of lifelong learning and the international guidance published by learned societies [10].

Adapting policies and regulation based on workforce intelligence may facilitate CPD activities intake by pharmacists, and contribute to further development of the profession [31].

Authors would like to express that further research is needed in the area, especially regarding evaluation of the CPD program with regard to the impact of learning outcomes and knowledge and skills development, thus meeting identified gaps through a needs analysis. Further evaluation will be needed to assess—on a basis of continuing evaluation and quality assurance—and monitor if stakeholders training offer is being tailored to pharmacists needs.

## Limitations and strengths

The main strength of this study consists in the exhaustiveness of data, since the Portuguese Pharmaceutical Society is the only authority where all active pharmacists need to be registered in the country, thus creating a centralized register. Since all active pharmacists are enrolled in the CPDp as a mandatory program, all educational activities are registered and are accounted for their professional development.

Because the CPD program is mandatory—all active pharmacists must be enrolled in the 5-year cycles—the study covers pharmacists with an extensive age range (28 to 90+ years old), and across all areas of pharmacy practice (community, hospital, clinical biology, industry, regulatory affairs, pharmaceutical distribution, military, among others). This fact could also impact and bias the analysis, as quite often recently graduated pharmacists invest in continuing education, rather than already established pharmacists, and the type of activities enrolled can be substantially different [3, 13, 26].

Analysis concerning CPD points should be made carefully, since there were changes regarding evaluation of CPD activities throughout the years. For example, before 2014, activities with or without evaluation were granted CPD points in the same rates (i.e., depending only on their duration). After 2014, the accredited CPD points depended on the presence or absence of evaluation, being activities with evaluation granted higher CPD points.

The study focused only on the activities that were accredited by the PPS by stakeholders (which was the main objective of this study). However, pharmacists can submit individual activities, but these represent a small proportion of the activities accounted for professional development.

Educational activities sponsored by stakeholders and accredited by the PPS represent the majority of activities that are participated by pharmacists in the overall CPD activities that are widely available for pharmacists. The quality assurance standards of the PPS are widely recognized, and accreditation outcome can severely impact positively pharmacists’ participation in activities.

## Conclusion

The study shows for the first time, a significant increase in the interest of CPD activities submitted for accreditation of the PPS throughout the period 2009–2019, yet a link between CPD and the professional area of practice was not identified. To overcome this, a revision of the CPD program was performed, and gradually came into force during 2019–2020, including the distinction between “core” (directly related to professional practice) and “non-core/satellite” (not directly linked with pharmaceutical practice but relevant to professional development) activities for the professional area.

Stakeholders who contributed more to accredited activities were sectoral associations, consultancy/training companies, the PPS, and pharmaceutical industry. Pharmacists still prefer face-to-face trainings; however, e-learning opportunities were already showing a trend towards increased interest, even in a pre-pandemic scenario.

Research suggests that there is still room for improvement for Academia to play a more interventive role in the lifelong learning education of Portuguese pharmacists, and further interactions are needed with Academia to understand how to better overcome any existing limitations.

## Supplementary Information

**Additional file 1: **Labels and classifications.

## Data Availability

The datasets used and/or analyzed during the current study are available from the corresponding author on reasonable request.

## Abbreviations

PPS: Portuguese Pharmaceutical Society
CPD: Continuous Profession Development
FIP: International Pharmaceutical Federation
EU: European Union
EFTA: European Free Trade Association
CPDp: Continuous Professional Development program
SD: Standard deviation
IQR: Inter-quartile range
