# Supplementary material for: A review of the continuous professional development system for pharmacists
Source: Hum Resour Health. 2022 Jan 6;20:3. doi: 10.1186/s12960-021-00700-1 (PMC8734236; doi:10.1186/s12960-021-00700-1)
Supplement: Supplementary file 1 — Additional file 1: Labels and classifications. [file 12960_2021_700_MOESM1_ESM.docx]

**Additional file 1**

| Annex S1 – Labels and classifications | | |  |
| --- | --- | --- | --- |
| Type of promoter | Geographic Scope | Area of Professional Focus | Type of Activity |
| 1. professional and sectoral associations,  2. national authorities,  3. consultancy/training companies,  4. wholesalers’ consortia,  5. students’ associations,  6. Academia,  7. pharmaceutical industry,  8. non-governmental organizations,  9. Portuguese Pharmaceutical Society,  10. hospitals and healthcare units | 1. national 2. international | 1. professional access  2. counselling  3. clinical biology  4. antibiotic therapy  5. regulatory affairs  6. biologic medicines  7. biosimilar medicines  8. natural sciences  9. pharmaceutical sciences  10. contraception  11. cosmetics / dermocosmetics  12. pharmaceutical care  13. dermatology  14. diabetes  15. medical devices  16. pharmaceutical supply chain/distribution  17. infectious diseases  18. rare diseases  19. pain  20. clinical trials  21. epidemiology  22. scientific studies  23. hospital pharmacy  24. pharmacokinetics  25. pharmacoeconomics  26. pharmacoepidemiology  27. pharmacology and pharmacotherapy  28. pharmaceutical technology and compounding  29. pharmacovigilance  30. wounds  31. phytotherapy  32. genetics  33. management and administration  34. law  35. veterinary medicines  36. nutrition  37. oncology  38. other topics  39. health education and promotion  40. pediatrics & childcare  41. quality assurance  42. chemistry  43. radiopharmaceuticals  44. healthcare and wellbeing  45. mental health  46. public health  47. basic life support / first aid  48. soft-skills  49. food supplements  50. alternative therapies  51. vaccines / injectable drugs  52. sales & marketing | 1. face-to-face trainings 2. e-learning trainings 3. conference 4. congress 5. post-graduation course 6. research studies 7. Specialist title 8. position held in profession association 9. master’s degree |
